# High-Contrast and Scattering-Type Transflective Liquid Crystal Displays Based on Polymer-Network Liquid Crystals

**DOI:** 10.3390/polym12040739

**Published:** 2020-03-26

**Authors:** Cheng-Kai Liu, Wei-Hsuan Chen, Chung-Yu Li, Ko-Ting Cheng

**Affiliations:** Department of Optics and Photonics, National Central University, Taoyuan 32001, Taiwan; aerobert007@gmail.com (C.-K.L.); lancelot0914@gmail.com (W.-H.C.); ga2007252655@gmail.com (C.-Y.L.)

**Keywords:** transflective liquid crystal displays, liquid crystals, polymer networks, linearly polarized light, light scattering

## Abstract

The methods to enhance contrast ratios (CRs) in scattering-type transflective liquid crystal displays (ST-TRLCDs) based on polymer-network liquid crystal (PNLC) cells are investigated. Two configurations of ST-TRLCDs are studied and are compared with the common ST-TRLCDs. According to the comparisons, CRs are effectively enhanced by assembling a linear polarizer at the suitable position to achieve better dark states in the transmissive and reflective modes of the reported ST-TRLCDs with the optimized configuration, and its main trade-off is the loss of brightness in the reflective modes. The PNLC cell, which works as an electrically switchable polarizer herein, can be a PN-90° twisted nematic LC (PN-90° TNLC) cell or a homogeneous PNLC (H-PNLC) cell. The optoelectric properties of PN-90° TNLC and those of H-PNLC cells are compared in detail, and the results determine that the ST-TRLCD with the optimized configuration using an H-PNLC cell can achieve the highest CR. Moreover, no quarter-wave plate is used in the ST-TRLCD with the optimized configuration, so a parallax problem caused by QWPs can be solved. Other methods for enhancing the CRs of the ST-TRLCDs are also discussed.

## 1. Introduction

Polymer-network (PN) liquid crystals (PNLCs) have attracted considerable attention because they can be electrically switched between optically transparent and scattering (translucent) states. Polymer fibrils are formed along local LCs in PNLCs to form PN during photopolymerization and usually can strongly anchor the surrounding LCs. PNLCs fabricated in a homogeneously (vertically) aligned LC cell can be used to develop a polarization-dependent (polarization-independent) device [1,2]. PNLCs can also be widely applied to various optical devices, such as light modulators, displays, and smart windows. [1,2,3,4,5,6,7,8,9,10,11]. PN-90° twisted nematic LC (PN-90° TNLC) cells have also extensively been studied [12,13,14,15,16,17]. One of the most interesting properties of a PN-90° TNLC cell is its asymmetrical transmission of linearly polarized light (LPL).

**Figure 1:**
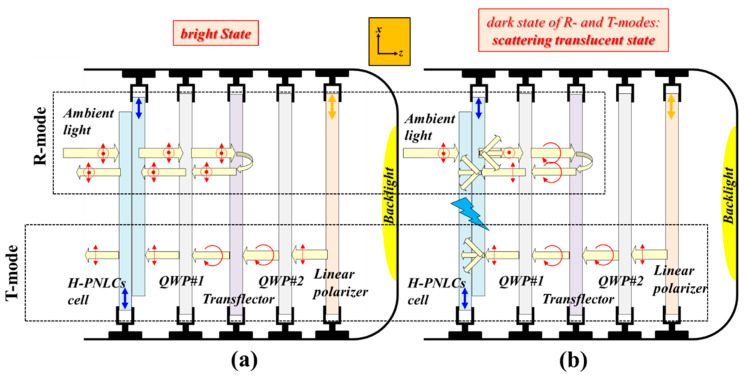
Schematics of a common scattering-type transflective liquid crystal displays (ST-TRLCDs) using a homogeneous polymer-network liquid crystal (H-PNLC) cell in (**a**) voltage-off bright (R- and T-modes) states and (**b**) voltage-on scattering-translucent (R- and T-modes) states [4]. QWP represents a quarter-wave plate. The “light blue thunder” symbol represents that the H-PNLC cell is being applied with a suitable electric field. Blue arrows plotted in H-PNLC cells and orange arrows plotted in linear polarizers represent the rubbing directions of the two substrates of an H-PNLC cell and the transmissive axes of linear polarizers, respectively; red arrows and dots represent the polarization states of linearly polarized light (LPL); circular arrows represent circularly polarized light.

Transmissive LC display (T-LCD) is a common technology that has been widely applied to various electronics globally. A backlight unit is assembled into a T-LCD to provide a light source for displaying images. The main drawback of T-LCDs is that the contrast ratio (CR), which is defined as the ratio of the luminance of output light in the bright state to that in the dark state, is relatively low under strong ambient light due to undesired glares. However, reflective LCDs (R-LCDs), which use ambient light as the sole light source to display images, possess relatively high CRs under strong ambient light but are inoperative under dim ambient light. To realize LCDs with high CR under strong and/or dim ambient light environments, several types of transflective LCDs (TRLCDs) are developed. A pixel region of a common TRLCD is usually divided into transmissive (T-) and reflective (R-) regions to work as T- and R-modes, respectively [1,4,18,19,20,21,22,23,24,25,26]. Figure 1 presents the structures of a common scattering-type TRLCD (ST-TRLCD), which consists of two quarter-wave plates (QWP#1 and QWP#2), a homogeneous PNLC (H-PNLC) cell, a linear polarizer, and a transflector [4]. One pixel of an ST-TRLCD, which does not need to be divided into two regions, works as the T- and R-modes simultaneously. The transmissive axis of the linear polarizer is along the *x*-axis. LPL with its polarization direction parallel to the *x*-axis remains unchanged after passing through the QWP#1 and QWP#2 when the optical axes of the QWP#1 and QWP#2 are perpendicular to each other, and the angle made between the optical axis of the QWP#2 and the polarization direction of the *x*-LPL is 45°. LCs in an H-PNLC cell are along the *x*-axis, allowing the cell applied with a suitable electric field to scatter LPL with its polarization direction parallel to the *x*-axis. The operation of the common ST-TRLCD (Figure 1) requires only one polarizer, so it can achieve high brightness. The bright states of the T- and R-modes of the common ST-TRLCD (Figure 1a) are realized in the absence of electric fields applied to the H-PNLC cell. Figure 1b illustrates that when the H-PNLC cell is applied with a suitable electric field, the T- and R-modes approach their scattering-translucent states, which are not good dark states. Thus, the CR of the common ST-TRLCD depicted in Figure 1 is insufficient for practical applications. Moreover, the use of the QWP close to the linear polarizer results in a parallax problem in the common ST-TRLCD [4]. Overall, to enhance the CR and to solve the parallax problem caused by QWPs, we redesign the optical components and remove the two QWPs based on the configuration of the common ST-TRLCDs.

This paper investigates the techniques to enhance CRs in ST-TRLCDs based on a PNLC cell. Two configurations of ST-TRLCDs are studied and are compared with the common ST-TRLCDs. According to the comparison, CR can be increased by assembling a linear polarizer at a suitable position in the reported ST-TRLCDs with the optimized configuration, because the light modulated by the scattering LC devices in T- and R-modes must pass through the linear polarizer to achieve better dark states, and its main trade-off is the loss of brightness in the R-modes. The adopted PNLCs, which work as electrically switchable polarizers, can be a PN-90° TNLC cell or an H-PNLC cell. The optoelectric performances of the PN-90° TNLC cell and those of the H-PNLC cell are compared, and the analyzed results determine that the reported ST-TRLCD with the optimized configuration using an H-PNLC cell can obtain the highest CR. Furthermore, no QWP is employed in the ST-TRLCD with the optimized configuration so that the parallax problem resulting from the QWP can be resolved. Other methods for enhancing the CRs of the reported ST-TRLCDs are also discussed.

## 2. Materials and Cell Fabrications

The fabrications of PN-90° TNLC and H-PNLC cells are elucidated here. NLCs, E7 (~95 wt %, Merck), and RM257 monomer (~5 wt %, Merck) were homogeneously mixed. The extraordinary refractive index (*n_e_*) and ordinary refractive index (*n_o_*) of E7 (RM257) are 1.73 and 1.519 (1.687 and 1.508), respectively. Two commercial empty cells with a 5 μm-thick cell gap (Instec Inc.) were filled with the homogeneous mixture. The top and bottom substrates of the cells were coated with planar alignment layers and treated with mechanical rubbing. The rubbing directions of one of the LC cells were orthogonal, while those of the other cells were parallel to each other. Afterward, the prepared LC cells were illuminated with UV light with an intensity of approximately 10 mW/cm^2^ for ~120 min to form the smooth PN gradually. Photo-initiator was not used herein to ensure the PN could be formed smoothly along the orientation of local LCs [16,27]. The cell treated with orthogonal (parallel) mechanical rubbing was named as the PN-90° TNLC (H-PNLC) cell.

## 3. Two Configurations of the Reported ST-TRLCDs

**Figure 2:**
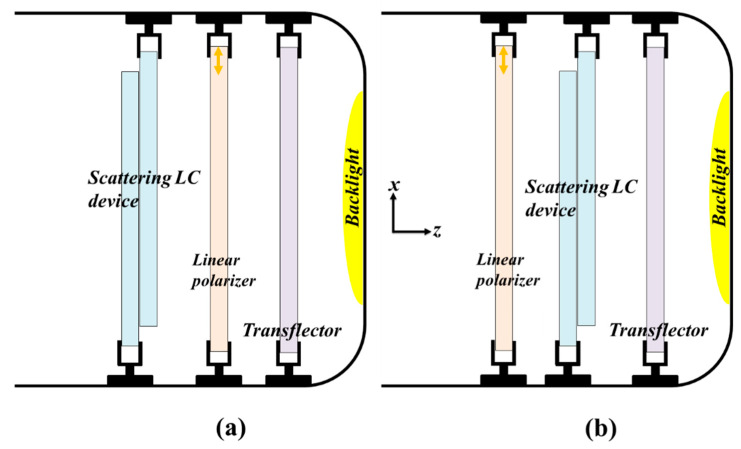
Configurations of (**a**) ST-TRLCD#1 and (**b**) ST-TRLCD#2. Both comprise one scattering liquid crystal (LC) device, one linear polarizer, and one transflector. The main difference in configuration between both is the arrangement of the linear polarizer and the scattering LC device. Orange arrows plotted in the linear polarizers represent their transmissive axes.

The strategy to enhance the CRs of ST-TRLCDs is elucidated as follows. First, the CRs of the two configurations of the reported ST-TRLCDs and the common ST-TRLCDs are compared to each other. The comparisons determine the configuration of the reported ST-TRLCD having the highest CR. The discussion will be given in Section 4.1. Second, the optoelectric performances of the PN-90° TNLC cell and those of the H-PNLC cell are compared to determine which one can further enhance the CR of the configuration of the reported ST-TRLCD having the highest CR. The discussion will be given in Section 4.2. Figure 2a,b shows the two configurations which comprise one scattering LC device, one linear polarizer, and one transflector [1,4]. The scattering LC device reported herein is an H-PNLC cell or a PN-90° TNLC cell. The difference in configuration between Figure 2a,b is the arrangement of the linear polarizer and the scattering LC device. Both transmissive axes of the linear polarizers adopted in Figure 2a,b are along the *x*-axis (orange arrow). The transflectors used in the configurations of Figure 2a,b reflect half of the intensity of the incident light. The two ST-TRLCDs displayed in Figure 2a,b are named as ST-TRLCD#1 and ST-TRLCD#2, respectively.

**Figure 3:**
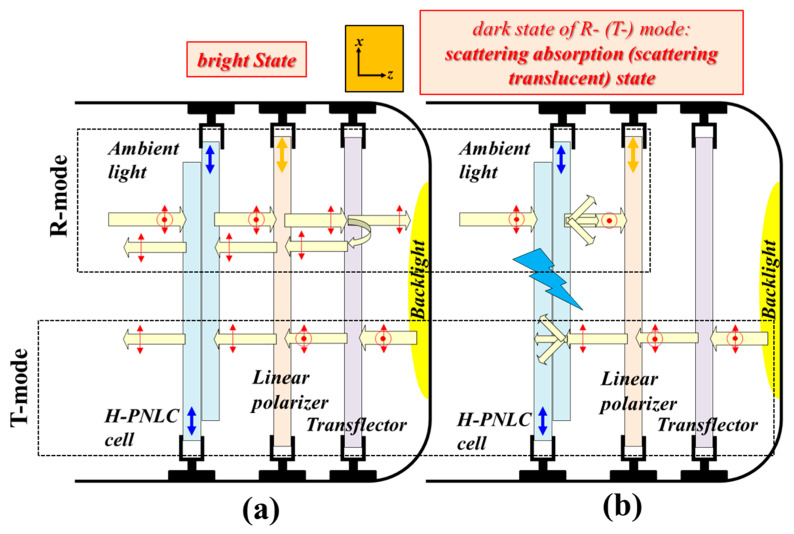
Configurations of a pixel of the ST-TRLCD#1 using an H-PNLC cell in (**a**) voltage-off bright (R- and T-modes) states and (**b**) voltage-on scattering-absorption (R-mode) and voltage-on scattering-translucent (T-mode) states. The “light blue thunder” symbol represents that the H-PNLC cell is being applied with a suitable electric field. Blue arrows plotted in H-PNLC cells and orange arrows plotted in linear polarizers represent the rubbing directions of the two substrates of an H-PNLC cell and the transmissive axes of linear polarizers, respectively; red arrows and dots represent the polarization states of LPL.

**Figure 4:**
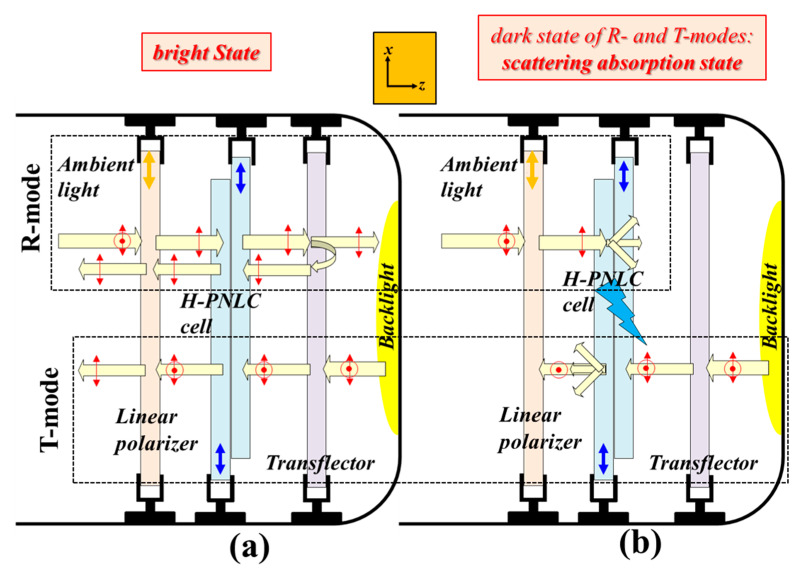
Configurations of a pixel of the ST-TRLCD#2 using an H-PNLC cell in (**a**) voltage-off bright (R- and T-modes) state and (**b**) voltage-on scattering-absorption (R- and T-modes) states. The “light blue thunder” symbol represents that the H-PNLC cell is being applied with a suitable electric field. Blue arrows plotted in H-PNLC cells and orange arrows plotted in linear polarizers represent the rubbing directions of the two substrates of an H-PNLC cell and the transmissive axes of linear polarizers, respectively; red arrows and dots represent the polarization states of LPL.

An H-PNLC cell is first adopted as a scattering LC device for demonstrating the two configurations of the reported ST-TRLCDs. Figure 3a,b (Figure 4a,b) exhibits the configuration of a pixel of the reported ST-TRLCD#1 (ST-TRLCD#2) in the bright and dark states, respectively, when the adopted scattering LC device is an H-PNLC cell. The dark states of the R- and T-modes shown in Figure 3b are scattering-absorption and scattering-translucent states, respectively. Both the dark states of the R- and T-modes shown in Figure 4b are scattering-absorption states. Blue arrows in the H-PNLC cell represent the rubbing directions of the two substrates of the H-PNLC cell, so the H-PNLC cells with LC directors parallel to *x*-axis, illustrated in Figure 3b and Figure 4b, applied with suitable electric fields scatter LPL with its polarization direction parallel to the *x*-axis when the incident LPL travels along the +*z*-axis or −*z*-axis. For a clear understanding, the elucidation of operation of the T- and R-modes shown in Figure 3 and Figure 4 are separated; a single pixel for these configurations can work as T- and R-modes simultaneously. The scattering-absorption state is defined when the light scattered by the H-PNLC cell applied with a suitable electric field is absorbed by the linear polarizer.

## 4. Results and Discussion

The discussions include seven parts. In Section 4.1.1 and Section 4.1.2, the operation principles of the reported ST-TRLCD#1 using an H-PNLC cell (Figure 3) and ST-TRLCD#2 using an H-PNLC cell (Figure 4) are elucidated, respectively; in Section 4.1.3, the CRs of the common ST-TRLCD (Figure 1), the reported ST-TRLCD#1 using an H-PNLC cell (Figure 3), and the reported ST-TRLCD#2 using an H-PNLC cell (Figure 4) are compared, and the comparisons confirm the configuration of the reported ST-TRLCD#2 using an H-PNLC cell (Figure 4) having the highest CR. In Section 4.1, we assume the H-PNLC cells can fully scatter the incident *x*-LPL, and let the incident *y*-LPL fully pass; incident *x*- or *y*-LPL can fully pass the H-PNLC cell without applying any electric field. In Section 4.2.1, the operation principle of ST-TRLCD#2 using an H-PNLC cell (Figure 4) is discussed by replacing the H-PNLC cell with the PN-90° TNLC cell; in Section 4.2.2, the optoelectric performances of the PN-90° TNLC and those of H-PNLC cells are investigated to examine which one can further enhance the CR of the configuration of the reported ST-TRCLCD#2 (Figure 2b). In Section 4.2.3, the results described in Section 4.2.2 are further analyzed theoretically. In Section 4.3, other methods to increase CR are discussed.

### 4.1. Evaluation of CRs of the Common ST-TRLCDs and the Two SR-TRLCDs Using H-PNLC Cells

#### 4.1.1. Operation Principle of the ST-TRLCD#1 Using an H-PNLC Cell

Referring to the R-mode of the reported ST-TRLCD#1 using an H-PNLC cell displayed in Figure 3a, the unpolarized ambient light remains unchanged after passing through the H-PNLC cell without applying any electric field, and is subsequently linearly polarized to *x*-LPL after passing through the linear polarizer. The *x*-LPL is then partially reflected by the transflector and loses half of its intensity. The reflected *x*-LPL passes through the linear polarizer and the H-PNLC cell without applying any electric field to approach the bright state of the R-mode. Referring to the T-mode of the reported ST-TRLCD#1 using an H-PNLC cell presented in Figure 3a, the unpolarized backlight is initially partially reflected by the transflector and loses half of its intensity. The remaining unpolarized light is linearly polarized to *x*-LPL after passing through the linear polarizer; the *x*-LPL then passes through the H-PNLC cell without applying any electric field to approach the bright state of the T-mode.

Referring to the R-mode of the reported ST-TRLCD#1 using an H-PNLC cell illustrated in Figure 3b, the unpolarized ambient light is scattered by the H-PNLC cell applied with a suitable electric field. The exiting *y*-LPL and the scattered light are then fully and partially absorbed by the linear polarizer, respectively. The polarization direction of the remaining scattered light linearly polarized by the linear polarizer is along the *x*-axis, and the light is then partially reflected by the transflector causing the loss of half of its intensity. The scattered light reflected by the transflector fully passes through the linear polarizer and is scattered by the H-PNLC cell applied with a suitable electric field again. This is the dark state of the R-mode; the detailed description of the scattered light after passing through the linear polarizer is not drawn in Figure 3b. Referring to the T-mode illustrated in Figure 3b, the unpolarized backlight is first partially reflected by the transflector and loses half of its intensity. The remaining unpolarized light is linearly polarized to *x*-LPL after passing through the linear polarizer and subsequently scattered by the H-PNLC cell applied with a suitable electric field to approach the dark state of the T-mode.

#### 4.1.2. Operation Principle of the ST-TRLCD#2 Using an H-PNLC Cell

Referring to the R-mode of the reported ST-TRLCD#2 using an H-PNLC cell depicted in Figure 4a, the unpolarized ambient light is linearly polarized to *x*-LPL after passing through the linear polarizer; the *x*-LPL then passes through the H-PNLC cell without applying any electric field. The *x*-LPL is subsequently partially reflected by the transflector and loses half of its intensity. The reflected *x*-LPL then passes through the H-PNLC cell without applying any electric field and the linear polarizer to approach the bright state of the R-mode. Referring to the T-mode of the reported ST-TRLCD#2 using an H-PNLC cell depicted in Figure 4a, the unpolarized backlight is first partially reflected by the transflector and loses half of its intensity. The remaining unpolarized light remains unchanged after passing through the H-PNLC cell without applying any electric field, and subsequently linearly polarized to *x*-LPL after passing through the linear polarizer to approach the bright state of the T-mode.

Referring to the R-mode of the reported ST-TRLCD#2 using an H-PNLC cell exhibited in Figure 4b, the unpolarized ambient light is linearly polarized to *x*-LPL after passing through the linear polarizer. The *x*-LPL is then scattered by the H-PNLC cell applied with a suitable electric field. The scattered light is then partially reflected by the transflector and loses half of its intensity. The scattered light reflected by the transflector comprises components of *x*- and *y*-LPLs. The scattered light reflected by the transflector having components of *x*-LPLs is subsequently scattered by the H-PNLC cell applied with a suitable electric field and eventually partially absorbed by the linear polarizer, and the scattered light reflected by the transflector having components of *y*-LPLs passes through the H-PNLC cell applied with a suitable electric field and is absorbed by the linear polarizer. This is the dark state of the R-mode; the detailed description of the scattered light exiting the H-PNLC cell applied with a suitable electric field is not drawn in Figure 4b. Referring to the T-mode displayed in Figure 4b, the unpolarized backlight is first partially reflected by the transflector and loses half of its intensity. The scattered light exiting the H-PNLC cell applied with a suitable electric field comprises components of *x*- and *y*-LPLs, and the former can be scattered by the H-PNLC cell applied with a suitable electric field. Accordingly, the *y*-LPL and the scattered light exiting the H-PNLC cell applied with a suitable electric field are then fully and partially absorbed by the linear polarizer to approach the dark state of the T-mode.

#### 4.1.3. Evaluation of CRs of the Three SR-TRLCDs

The CRs of the three ST-TRLCDs, including the common ST-TRLCD (Figure 1), the reported ST-TRLCD#1 using an H-PNLC cell (Figure 3), and the reported ST-TRLCD#2 using an H-PNLC cell (Figure 4), are compared with each other. The dark states of the T- and the R-modes of the common ST-TRLCDs (Figure 1) are in scattering-translucent states when the H-PNLC cell is applied with a suitable electric field. When the H-PNLC cell is applied with a suitable electric field, the dark states of the T- and R-modes of the ST-TRLCD#1 using an H-PNLC cell (Figure 3b) are considered the scattering-translucent and scattering-absorption states, respectively; the dark states of the T- and R-modes of the ST-TRLCD#2 using an H-PNLC cell (Figure 4b) are considered the scattering-absorption states. To quantitatively discuss the dark state performances of both modes of the three ST-TRLCDs, we assume that (i) the intensity of unpolarized light passing through the linear polarizer is 50% reduced, (ii) the polarization of the *x*-LPL traveling along −*z*-axis after being scattered by the H-PNLC cell applied with a suitable voltage is considered unpolarized, and the intensity of the scattered light is *α* reduced (*α* < 1), (iii) the polarization of the *x*-LPL, traveling along +*z*-axis after being scattered by the H-PNLC cell applied with a suitable voltage is considered unpolarized, and the intensity of the scattered light is *β* reduced (*α* < *β* < 1), (iv) *y*-LPL can fully pass through the H-PNLC cell applied with a suitable voltage, and (v) the reduction of the intensity of any light passing through the QWP#1 or the QWP#2 is 4% due to reflection, absorption, etc. The H-PNLC cells in the three ST-TRLCDs are identical, and the initial intensities of backlight and ambient light sources are assumed to be one. The value *β* depends on the distance between the H-PNLC cell and the transflector. For simplicity, we assume the values of *β* of common ST-TRLCDs (Figure 1), the reported ST-TRLCD#1 using an H-PNLC cell (Figure 3), and the reported ST-TRLCD#2 using an H-PNLC cell (Figure 4) are similar. The value *α* is smaller than the value *β* because the distance between users and the ST-TRLCDs is far larger than the distance between the H-PNLC cell and the transflector. Table 1 shows the comparisons of dark state performances (output of the dark state) of both T- and R-modes of the three ST-TRLCDs. Referring to Table 1, the scattering-absorption states of both modes of the ST-TRLCD#2 using an H-PNLC cell (Figure 4b) and R-mode of the ST-TRLCD#1 using an H-PNLC cell (Figure 3b) possess better dark performance than the scattering-translucent states of the R-/T-mode of the common ST-TRLCDs (Figure 1). Dark state performance is more important than the bright state one when it comes to CRs [1,4,28,29]. Overall, Table 1 shows that the values of the output of the dark states of T- and R-modes of the ST-TRLCD#2 using an H-PNLC cell (Figure 4b) are the lowest among the three values of the outputs of the dark states of T- and R-modes, respectively, so the CRs of the T- and R-modes of ST-TRLCD#2 using an H-PNLC cell (Figure 4) are the highest CRs among the three ST-TRLCDs.

The trade-off of the use of the reported ST-TRLCD#2 using an H-PNLC cell (Figure 4) are discussed. The reflectance of the bright state of R-mode in the ST-TRLCD#2 using an H-PNLC cell illustrated in Figure 4 is theoretically weaker than that in the common ST-TRLCD depicted in Figure 1. This is reasonable because the ambient light for the R-mode depicted in Figure 4 passes through the linear polarizer twice and loses half of its intensity, and the ambient light for the R-mode in Figure 1 passes through two QWPs rather than any linear polarizer. The loss is much higher in light intensity that passes through the linear polarizer twice than that through the QWP twice.

### 4.2. Comparison of CRs of ST-TRLCD#2 Using H-PNLC and PN-90° TNLC Cells

#### 4.2.1. Operation Principle of the ST-TRLCD#2 When the Scattering LC Device is a PN-90° TNLC Cell

**Figure 5:**
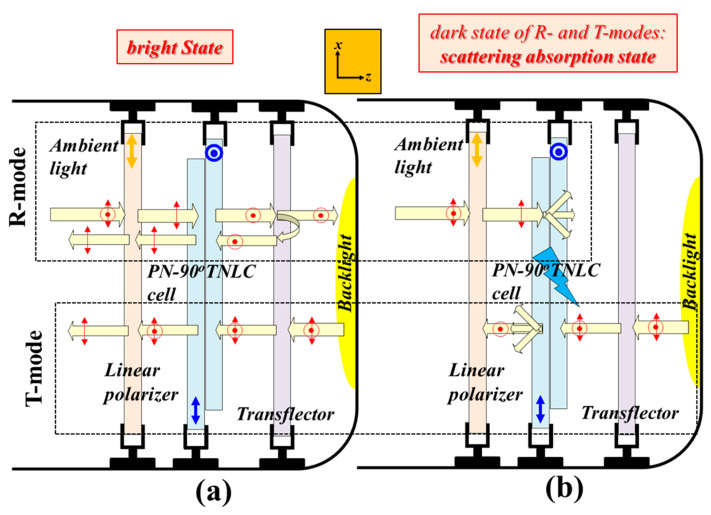
Configurations of a pixel of the ST-TRLCD#2 when the H-PNLC cell is replaced by a PN-90° TNLC cell in (**a**) voltage-off bright (R- and T-modes) state and (**b**) voltage-on scattering-absorption (R- and T-modes) states. The “light blue thunder” symbol represents that the PN-90° TNLC cell is being applied with a suitable electric field. Blue arrows and dots shown in PN-90° TNLC cells and orange arrows plotted in linear polarizers represent the rubbing directions of the two substrates of a PN-90° TNLC cell and the transmissive axes of linear polarizers, respectively; red arrows and dots represent the polarization states of LPL.

Figure 5 shows the reported ST-TRLCD#2 when the scattering LC device plotted in Figure 2b is a PN-90° TNLC cell. The rubbing direction of the substrate of the PN-90° TNLC cell close to the linear polarizer (transflector) is along the *x*- (*y*-) axis. The blue arrows and dots represent the rubbing directions of the substrates. Referring to the R-mode depicted in Figure 5a, the unpolarized ambient light is linearly polarized to *x*-LPL after passing through the linear polarizer. The *x*-LPL, passing through the PN-90° TNLC cell without applying any electric field, becomes *y*-LPL, and is subsequently partially reflected by the transflector, thereby losing half of its intensity. The reflected *y*-LPL, passing through the PN-90° TNLC cell, becomes *x*-LPL, and eventually passes through the linear polarizer to approach the bright state of the R-mode. Referring to the T-mode depicted in Figure 5a, the unpolarized backlight is first partially reflected by the transflector, thereby losing half of its intensity. The remaining unpolarized light remains unchanged after passing through the PN-90° TNLC cell without applying any electric field, and is subsequently linearly polarized to *x*-LPL after passing through the linear polarizer to approach the bright state of the T-mode.

Referring to the R-mode exhibited in Figure 5b, the unpolarized ambient light is linearly polarized to *x*-LPL after passing through the linear polarizer. The *x*-LPL is then scattered by the PN-90° TNLC cell applied with a suitable electric field. The scattered light is partially reflected by the transflector, thereby losing half of its intensity. The scattered light reflected by the transflector having component of *y*-LPL is subsequently scattered by the PN-90° TNLC cell applied with the suitable electric field, and then partially absorbed by the linear polarizer; the scattered light reflected by the transflector having component of *x*-LPL partially passes through the PN-90° TNLC cell, and is absorbed by the linear polarizer due to polarization rotation of PN-90° TNLC cell; the detailed description of the scattered light exiting the PN-90°-TNLC cell is not drawn in Figure 5b. This is the dark state of the R-mode. Referring to the T-mode displayed in Figure 5b, the unpolarized backlight is first partially reflected by the transflector, thereby losing half of its intensity. The PN-90° TNLC cell applied with a suitable electric field scatters *y*-LPL; at the same time, the *x*-LPL can partially transmit the PN-90° TNLC cell, and its polarization direction is rotated 90° to become *y*-LPL after passing through the PN-90° TNLC cell. The *y*-LPL and the scattered light exiting the PN-90° TNLC cell applied with a suitable electric field are fully and partially absorbed by the linear polarizer, respectively, to approach the dark state of the T-mode.

The dark states of the T-/R-mode plotted in Figure 4b and Figure 5b are the scattering-absorption states. To determine which state is better, we compared the electro-optical properties of the PN-90° TNLC and H-PNLC cells in Section 4.2.2 and Section 4.2.3. The LC cell with better scattering performance can be adopted as the scattering LC device in the reported ST-TRLCD#2 (Figure 2b).

#### 4.2.2. Optoelectric Performances of PN-90° TNLC and H-PNLC Cells

**Figure 6:**
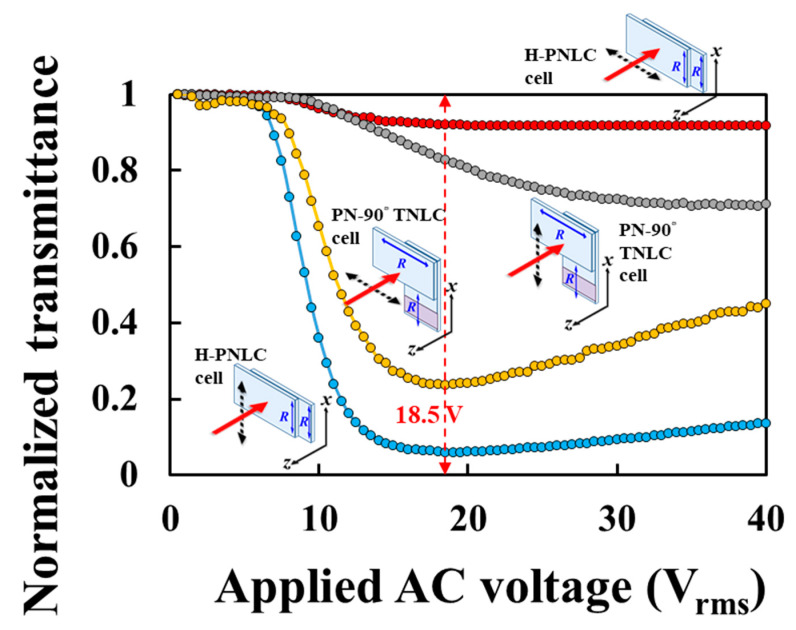
Transmittance as a function of the applied AC voltage curves of the H-PNLC and the PN-90° TNLC cells measured using an He–Ne laser with different polarization states. The applied AC field is a square-wave field with a frequency of 1 KHz. *R* represents the rubbing direction.

Figure 6 depicts the normalized transmittance as a function of the applied AC voltage (T–V) curves of the PN-90° TNLC cell and the H-PNLC cell. The probe beam was a He–Ne laser in two different LP states. The distance between the photodetector and the LC cell was set to be approximately 15 cm. The red and gray (blue and orange) curves in Figure 6 plot the T-V curves using a LP He–Ne laser with its linear polarization directions, marked as black dashed double-headed arrows, which were perpendicular (parallel) to the rubbing directions on the entrance planes of the substrates of the H-PNLC and PN-90° TNLC cells, respectively. All He–Ne lasers, whose propagation directions are plotted by red solid arrows, traveled along the −*z*-axis. No analyzer was placed in front of the photodiode.

The orange (blue) curve of Figure 6 shows that the minimum normalized transmittance of PN-the 90° TNLC (H-PNLC) cell reached ~0.24 (~0.059) when the applied voltage was 18.5 V_rms_ and the incident light was *y*-LPL (*x*-LPL). The result indicates the dark state performance of the H-PNLC cell applied with 18.5 V_rms_ for incident *x*-LPL is better than that of the PN-90° TNLC cell applied with 18.5 V_rms_ for incident *y*-LPL. On the other hand, the red (gray) curve of Figure 6 shows that the transmittance of light exiting the H-PNLC (PN-90° TNLC) cell applied with 18.5 V_rms_ is ~0.92 (~0.83) when the incident light is *y*-LPL (*x*-LPL); the results indicate that the amount of light, blocked by the linear polarizer plotted in Figure 4b for incident *y*-LPL, is higher than that blocked by the linear polarizer shown in Figure 5b for incident *x*-LPL, so this is another reason that the CR of T/R-mode of Figure 4b is higher than that of Figure 5b. Moreover, the reason caused the loss of transmittance of ~0.08 (0.17), shown as the red (gray) curve of Figure 6 when the applied voltage is 18.5 V_rms_ and the incident light is *y*-LPL (*x*-LPL), is that the incident light is scattered. Scattered light can partially pass through the linear polarizer, so more scattered light can pass through the linear polarizer in Figure 5b than that in Figure 4b to degrade CRs. The discussions will be further investigated in Section 4.2.3. Overall, the H-PNLC cell is preferred to be adopted in ST-TRLCD#2 (Figure 2b) to obtain high CR.

**Figure 7:**
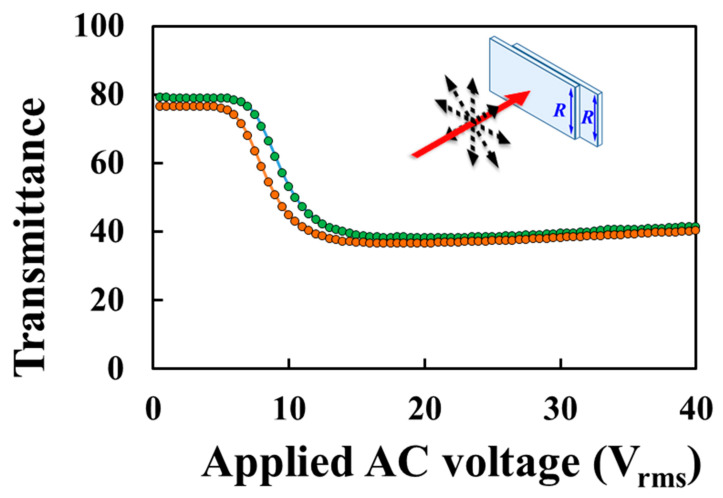
Hysteresis of the T–V curve of the H-PNLC cell measured by the unpolarized He–Ne laser. The applied AC field is a square-wave field with a frequency of 1 KHz.

**Figure 8:**
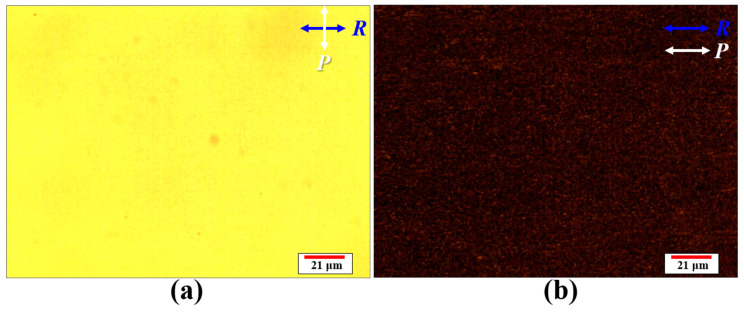
Microscopic images of the H-PNLC cell applied with 18.5 V_rms_ under the configurations consistent with those of (**a**) the red and (**b**) the blue curves in Figure 6 using a separate experiment. Blue (***R***) and white (***P***) arrows represent the rubbing directions of the two substrates of the H-PNLC cell and the polarization direction of the incident LP backlight of the microscope, respectively.

Figure 7 plots the hysteresis of the T–V curve of the H-PNLC cell measured using an unpolarized He–Ne laser (the red arrow shows the propagation direction, and the four black double-headed dashed arrows depict the unpolarized state). The green and orange curves represent the T–V curves obtained by increasing and decreasing the applied AC voltages stepwise, respectively. The hysteresis of the H-PNLC cell could be eliminated by doping specific dopants into the mixture [1,30]. The transmittance of incident unpolarized light passing through the H-PNLC cell without any applied voltage is ~79%, as illustrated in Figure 7. To enhance the maximum transmittance, the substrates can be coated with antireflection films [31]. Figure 8a,b shows the microscopic images of the H-PNLC cell applied with 18.5 V_rms_ under the configurations consistent with those of the red and blue curves plotted in Figure 6 using a separate experiment, respectively. The intensities of the backlight sources of the transmissive polarized optical microscopy for capturing the two images are the same. The brightness of Figure 8b is darker than that of Figure 8a due to light scattering. The observation corresponds to the normalized transmittances of red and blue curves in Figure 6 when the applied voltage is 18.5 V_rms_.

#### 4.2.3. Comparison of the Scattering of PN-90° TNLC and H-PNLC Cells

Based on Figure 6, scattering performance (SP) of H-PNLCs applied with 18.5 V_rms_, namely SP_H-PNLCs_, can be described by the following equation, in which the reflection caused by air–glass boundaries is ignored, when the incident light is unpolarized [16,32,33,34]:

(1) $$\begin{array}{c} SP_{H-PNLCs}=\frac{1}{2}\left\{\left[1-exp\left(-A_{x}\frac{\Delta n_{eff_{x}}^{2}}{\lambda_{0}^{2}}d\right)\right]+\left[1-exp\left(-A_{y}\frac{\Delta n_{eff_{y}}^{2}}{\lambda_{0}^{2}}d\right)\right]\right\}\\ =\frac{1}{2}\{\int_{0}^{d}A_{x}\frac{\Delta n_{eff_{x}}^{2}}{\lambda_{0}^{2}}exp\left(-A_{x}\frac{\Delta n_{eff_{x}}^{2}}{\lambda_{0}^{2}}h\right)dh\\ +\int_{0}^{d}A_{y}\frac{\Delta n_{eff_{y}}^{2}}{\lambda_{0}^{2}}exp\left(-A_{y}\frac{\Delta n_{eff_{y}}^{2}}{\lambda_{0}^{2}}h\right)dh\} \end{array}$$

where *h*, *A_x_, A_y_*, Δ*n_effx_*, Δ*n_effy_*, and *λ_o_* are, respectively, the positions in H-PNLC bulk, average domain size of multi-LC domains (mLCds) that the incident *x*-LPL encounters, average domain size of mLCds that the incident *y*-LPL encounters, average effective refractive index difference among different mLCds that the incident *x*-LPL encounters, average effective refractive index difference among different mLCds that the incident *y*-LPL encounters, and wavelength of incident light. Equation (1) assumes the sizes of the mLCds are comparable with visible light wavelengths, and the phase retardation caused by LCs in each mLCds is small [32]. The *h* value ranges from *0* to *d*, where *d* is the thickness of the LC cell, and *0* is defined as the position at the inner entrance plane of the substrate. Δ*n_effx_* and Δ*n_effy_* values are proportional to the LC birefringence (Δ*n*), which is dependent on the amplitude of the applied voltage and the selection of LCs [32]. The LCs are set to be perfectly rotated on the *xz*-plane by the applied voltage of 18.5 V_rms_. The incident *x*-LPL and *y*-LPL are scattered while traveling in the H-PNLC cell applied with the voltage of 18.5 V_rms_, and their polarization states remain unchanged. Overall, the value of the integral of $\int_{0}^{d}A_{y}\frac{\Delta n_{eff_{y}}^{2}}{\lambda_{0}^{2}}exp\left(-A_{y}\frac{\Delta n_{eff_{y}}^{2}}{\lambda_{0}^{2}}h\right)dh$ should be smaller than that of $\int_{0}^{d}A_{x}\frac{\Delta n_{eff_{x}}^{2}}{\lambda_{0}^{2}}exp\left(-A_{x}\frac{\Delta n_{eff_{x}}^{2}}{\lambda_{0}^{2}}h\right)dh$. The former (latter) integral represents the SP_H-PNLCs_ for the incident *y*- (*x*-) LPL after passing through the H-PNLC cell applied with the voltage of 18.5 V_rms_. Considering that the polarization states of *x*-LPL and *y*-LPL keep unchanged while traveling in the H-PNLC cell applied with the voltage of 18.5 V_rms_, they are fully scattered in each position in bulk. Theoretically, if all LCs are rotated on the *xz*-plane by the applied voltage of 18.5 V_rms_, the integral of $\int_{0}^{d}A_{y}\frac{\Delta n_{eff_{y}}^{2}}{\lambda_{0}^{2}}exp\left(-A_{y}\frac{\Delta n_{eff_{y}}^{2}}{\lambda_{0}^{2}}h\right)dh$ comes close to 0, because *y*-LPL incident light always encounters *n_o_* of LCs in all mLCds, and Δ*n_effy_* is close to 0. The minimum transmittance plotted as the blue curve of Figure 6 is not equal to 0, because the polymer fibrils do not actually grow perfectly along the local LCs, indicating that the LCs are not completely rotated on the *xz*-plane by the applied electric fields.

The polarization states of the *x*-LPL and *y*-LPL change to various elliptically polarized lights (EPLs) with different major and minor axes at different positions while traveling in the PN-90° TNLC cell applied with a voltage of 18.5 V_rms_ [16,17,35]. The components of EPLs with polarization that are parallel to the local LCs are scattered, whereas the components of EPLs with polarization that are perpendicular to the local LCs are not scattered because it encounters the *n_o_* of LCs in all mLCds. The adopted mixtures, the fabrication of UV photopolymerization, the fabrication of the empty cells, and cell thickness of the two LC cells are similar, and the main difference between the two cells is the different orientations of LCs in the PN-90° TNLCs and H-PNLCs. Hence, we believe that the qualitative discussions of the SP_H-PNLCs_ and SP_PN-90° TNLCs_ are still valid and can give useful information. The incident *x*-LPL and *y*-LPL are fully scattered in each position in the H-PNLC cell. However, the incident *x*-LPL (*y*-LPL) transforms into various EPLs in LC bulk. The components of EPLs with polarization that are perpendicular (parallel) to the local LCs are not (are) scattered. Hence, based on Figure 6, the SP_PN-90° TNLCs_ of incident *y*-LPL (*x*-LPL) should be lower (higher) than $A_{x}\frac{\Delta n_{eff_{x}}^{2}}{\lambda_{0}^{2}}exp\left(-A_{x}\frac{\Delta n_{eff_{x}}^{2}}{\lambda_{0}^{2}}h\right)dh$ [$A_{y}\frac{\Delta n_{eff_{y}}^{2}}{\lambda_{0}^{2}}exp\left(-A_{y}\frac{\Delta n_{eff_{y}}^{2}}{\lambda_{0}^{2}}h\right)dh$] in each position (*h*), except in position 0. Overall, based on Figure 6, the value of SP_H-PNLCs_ of incident *x*-LPL (*y*-LPL) should be higher (lower) than that of SP of PN-90° TNLC cell of incident *y*-LPL (*x*-LPL). Overall, the scattering performance of the H-PNLC cell is better than that of the PN-90° TNLC cell, so ST-TRLCD#2 using an H-PNLC cell (Figure 4) can achieve better CR than ST-TRLCD#2 using a PN-90° TNLC cell (Figure 5). The full operation of the ST-TRLCD#2 using an H-PNLC cell (Figure 4) has been reported in Section 4.1.2.

**Figure 9:**
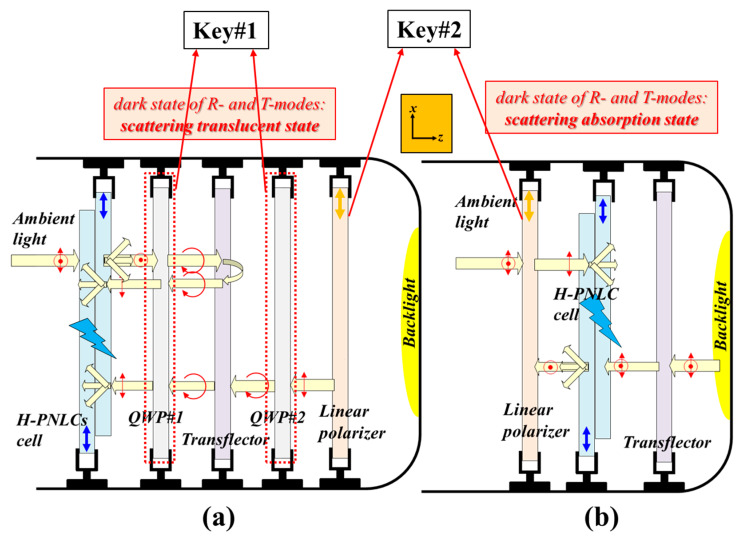
Comparisons between (**a**) the common ST-TRLCD (Figure 1b) and (**b**) the reported ST-TRLCD#2 (Figure 4b) using an H-PNLC cell. Key#1 and Key#2 are the elimination of QWPs and the rearrangement of linear polarizer, respectively.

Compared with the performances of T- and R-modes of the common ST-TRLCD (Figure 1b and Figure 9a), the improved performances of the reported ST-TRLCD#2 using an H-PNLC cell (Figure 4b and Figure 9b) result from Key#1 and Key#2, which are the removal of QWPs and the rearrangement of the linear polarizer, respectively. Regarding the bright state of the T-mode, the loss of the transmittance drawn in Figure 9b is slightly lower than that drawn in Figure 9a because the former has two QWPs to cause additional reflections. Regarding the dark state of the T-mode, the linear polarizer in Figure 9b absorbs the *y*-LPL and the *y*-component of scattering light exiting the H-PNLC cell applied with a suitable voltage, however, the linear polarizer in Figure 9a only absorbs *y*-LPL. Accordingly, the dark state and CR of T-mode shown in Figure 9b are better than those plotted in Figure 9a. Moreover, QWPs induced parallax problem does not exist in the ST-TRLCD#2 using an H-PNLC cell (Figure 4) due to Key#1. Regarding the dark state of the R-mode, the linear polarizer in Figure 9b absorbs *y*-LPL and the *y*-component of scattering light exiting the H-PNLC cell applied with a suitable voltage to realize better dark state and CR, while the linear polarizer in Figure 9a contributes nothing to the performance. Accordingly, the dark state and CR of R-mode shown in Figure 9b are better than those depicted in Figure 9a. The above discussions are considered in the calculations in Table 1 (see Appendix A).

### 4.3. Other Methods to Enhance CR in ST-TRLCD#2 Using an H-PNLC Cell

To further enhance the CR of the scattering-absorption state of the T- and R-modes of the reported ST-TRLCD#2 using an H-PNLC cell (Figure 4), the brightness of the backlight and ambient light can be reduced, respectively. Moreover, the reduced brightness will enhance other performances, such as color gamut and protection of eyes from damage by blue light, of the reported ST-TRLCD#2 using an H-PNLC cell (Figure 4). Functional films can be assembled to eliminate the undesirable light, which does not only reduce the brightness of the backlight but also expand the color gamut in T- and/or R-modes of the reported ST-TRLCD#2 using an H-PNLC cell (Figure 4) [36,37]. CR can also be enhanced by doping a small concentration of dichroic dyes into the H-PNLCs [1,4]. In addition, human eyes with long-term exposure to blue light can be damaged [38,39,40,41]. Therefore, the intensity of blue light from backlight sources can also be reduced to increase the CRs of LCDs and protect the eyes from potential damage at the same time.

## 5. Conclusions

The investigation to determine the ST-TRLCD with the best CR among the four ST-TRLCDs, including the common ST-TRLCD, the reported ST-TRLCD#1 using an H-PNLC cell, and the reported ST-TRLCD#2 using a PN-90° TNLC cell or H-PNLC cell, is discussed. The CRs in the T-/R-mode of the reported ST-TRLCD#2 using an H-PNLC cell (Figure 4) are higher than those in the common ST-TRLCDs (Figure 1), the reported ST-TRLCD#1 using an H-PNLC cell (Figure 3), and the reported ST-TRLCD#2 using a PN-90° TNLC cell (Figure 5). However, the reflectance in the R-mode of the reported ST-TRLCD#2 using an H-PNLC cell (Figure 4) is lower than that in the common ST-TRLCDs (Figure 1). We also qualitatively compared the optoelectric performance of the PN-90° TNLC and the H-PNLC cells. Moreover, no QWP is adopted in the ST-TRLCD#2 using an H-PNLC cell (Figure 4), so a parallax problem caused by QWPs is solved. Other methods for further enhancing the CR in the reported ST-TRLCD#2 using an H-PNLC cell presented in Figure 4 are discussed. The reported ST-TRLCD#2 using an H-PNLC cell (Figure 4) fabricated by an off-resonant light source and corresponding low absorption materials can be further investigated in the future [42,43,44]. The H-PNLC cell fabricated by copolymer network LCs (coPNLC) can also be investigated in this reported system [45,46,47]. Lorenz et al. reported that the operating voltage of the H-PNLC cell fabricated by coPNLCs with suitable concentrations of the mesogenic and the non-mesogenic monomers is around 8–12 V [46]. Other polymer materials can also be investigated for fabricating H-PNLC/coPNLC cells to decrease their operation voltages [1,2,10,48,49,50]. Overall, the optimized photopolymerization to fabricate the H-PNLC cell meeting the criteria for real display applications should be further investigated [1,3,4]. Moreover, the fabrication of the reported ST-TRLCD#2 using an H-PNLC cell (Figure 4) is considered simpler than that of common ST-TRLCDs (Figure 1).

## Figures and Tables

**Table 1:** Comparisons of dark state performances (output of the dark state) of both T- and R-modes of the three ST-TRLCDs (α < β < 1). For detailed calculations, refer to the Appendix A.

	Common ST-TRLCD		The Reported ST-TRLCD#1 Using an H-PNLC Cell (Figure 3b)		The Reported ST-TRLCD#2 Using an H-PNLC Cell (Figure 4b)	
	T-mode	R-mode	T-mode	R-mode	T-mode	R-mode
Output of the dark state	0.2304α	0.2304α +0.1152αβ +0.1152β	0.25α	0.125αβ	0.125α	0.0625αβ
	Scattering-translucent state			Scattering-absorption state		

## Appendix A

**Table A1:** The detailed calculations of the outputs of the dark states of the three ST-TRLCDs in Table 1 are listed below.

**Figure 1b**	**Output of the Dark state of a Common ST-TRLCD**
**T-mode**	Output = 1 × 0.5 × 0.96 × 0.5 × 0.96 × α = 0.2304α
**R-mode**	Output = [1 × 0.5 × 0.96 × 0.5 × 0.96 × α] + [1 × (0.5 × β) × 0.96 × 0.5 × 0.96 × (0.5 × α)] + [1× (0.5 × β) × 0.96 × 0.5 × 0.96 × 0.5] = 0.2304α + 0.1152αβ + 0.1152β
**Figure 3b**	**Output of the dark state of the reported ST-TRLCD#1 using an H-PNLC cell**
**T-mode**	Output = 1 × 0.5 × 0.5 × α = 0.25α
**R-mode**	Output = 1 × (0.5 × β) × 0.5 × 0.5 × α = 0.125αβ
**Figure 4b**	**Output of the dark state of the reported ST-TRLCD#2 using an H-PNLC cell**
**T-mode**	Output = 1 × 0.5 × (0.5 × α) × 0.5 = 0.125α
**R-mode**	Output) = 1 × 0.5 × β × 0.5 × (0.5 × α) × 0.5 = 0.0625αβ
